# Inflammation and Cell Death in Age-Related Macular Degeneration: An Immunopathological and Ultrastructural Model

**DOI:** 10.3390/jcm3041542

**Published:** 2014-12-22

**Authors:** Christopher P. Ardeljan, Daniel Ardeljan, Mones Abu-Asab, Chi-Chao Chan

**Affiliations:** 1 Histology Core, Laboratory of Immunology, National Eye Institute/National Institutes of Health, Bethesda, Maryland 20892-1857, MD, USA; E-Mails: christopher.ardeljan@nih.gov (C.P.A.); mones@nei.nih.gov (M.A.-A.); 2 Human Genetics Program, Johns Hopkins University School of Medicine, Baltimore, Maryland 21205, MD, USA; E-Mail: daniel.ardeljan@gmail.com; 3 Immunopathology Section, Laboratory of Immunology, National Eye Institute/National Institutes of Health, Bethesda, Maryland 20892-1857, MD, USA

**Keywords:** age-related macular degeneration (AMD), macrophage, microglia, inflammasome, cytokine, apoptosis, autophagy, necroptosis, pyroptosis

## Abstract

The etiology of Age-related Macular Degeneration (AMD) remains elusive despite the characterization of many factors contributing to the disease in its late-stage phenotypes. AMD features an immune system in flux, as shown by changes in macrophage polarization with age, expression of cytokines and complement, microglial accumulation with age, *etc.* These point to an allostatic overload, possibly due to a breakdown in self *vs.* non-self when endogenous compounds and structures acquire the appearance of non-self over time. The result is inflammation and inflammation-mediated cell death. While it is clear that these processes ultimately result in degeneration of retinal pigment epithelium and photoreceptor, the prevalent type of cell death contributing to the various phenotypes is unknown. Both molecular studies as well as ultrastructural pathology suggest pyroptosis, and perhaps necroptosis, are the predominant mechanisms of cell death at play, with only minimal evidence for apoptosis. Herein, we attempt to reconcile those factors identified by experimental AMD models and integrate these data with pathology observed under the electron microscope—particularly observations of mitochondrial dysfunction, DNA leakage, autophagy, and cell death.

## 1. Introduction

Age-related Macular Degeneration (AMD), a degenerative disease of the outer retina, is the leading cause of central irreversible blindness in the United States [1], accounting for 54% of all blindness in Americans of European ancestry, as well as 5% of all blindness globally [2]. While as much as 80% of all global blindness can be attributed to preventable or treatable causes such as cataracts and refractive errors, AMD presents a considerable challenge for the vision community because its etiology has not yet been clearly resolved and treatment options are limited. Data obtained from NHANES revealed that approximately 6.5% of the US population over age 40 shows clinical signs of AMD [3].

The eye is an immune privileged organ owing to the unique anatomic structure of the blood-retinal barrier composed of tight junctions, the lack of direct lymphatic drainage, and the interaction of Fas-FasL, which induces apoptosis of invading lymphocytes [4]. In addition, retinal pigment epithelial (RPE) cells have been shown to secrete factors that suppress an immune response, including TGF-β, somatostatin, thrombospondin, and pigment epithelium derived factor (PEDF) [5]. 

Much of the scientific literature on AMD has focused on markers of inflammation such as the inflammatory cytokines IL-1β, IL-18, and IL-17, as well as the complement system, the involvement of macrophages, and more recently, inflammasomes of the innate immune system. An important question that needs to be answered is whether inflammation is the root cause of AMD, or if the initial events are the result of metabolic abnormalities, hypoxia, and oxidative stress, with the resulting inflammation constituting only a secondary insult which is observed once the disease has progressed to intermediate AMD or late-stage phenotypes of geographic atrophy (GA) and choroidal neovascularization (CNV). In this review, we will summarize the current body of knowledge on the immunopathological contributors to AMD and discuss the evidence for each of these components in turn. We combine these advancements with our ultrastructural observations in order to present a synthesis of an AMD etiological model. We believe that the available molecular and ultrastructural data on AMD has permitted the construction of such a hypothesis. Our ultimate goal is to explore these avenues in order to facilitate the development of effective treatment and prevention tools.

## 2. Innate Immunity in AMD

The innate immune system comprises several levels of immediate protection and response to pathogens, including physical barriers, circulating proteins (largely the complement system), cells of the innate response such as monocytes and neutrophils, and pattern-recognition receptors, or PRRs, which are expressed by cells involved in innate immunity. The PRRs include Toll-like receptors (TLRs), RIG-I-like receptors (RLRs), NOD-like receptors (NLRs), and C-type lectin receptors (CLRs) [6].

### 2.1. Macrophage Phenotypes and Polarization

Macrophages are effector cells of the innate immune response whose diverse functions include phagocytosis and antigen presentation in the processes of both inflammation (subacute and chronic) and wound healing. Macrophages sense pathogen-associated molecular patterns (PAMPs) via PRRs expressed on the cell membrane and initiate the production of inflammatory cytokines and chemokines. Macrophages also act as sensors of danger in the host unrelated to microbial infection, responding to damage-associated molecular pattern molecules (DAMPs) as well. Endogenous danger signals released by stressed cells and damaged tissues trigger inflammation via TLRs and NLRs in macrophages, as well as the interleukin-1 receptor (IL-1R), just as PAMPs do. These DAMPs are released by stressed tissue and allow immune effectors to distinguish healthy-self from damaged-self, and include danger signals as diverse as heat-shock proteins, fibrinogen, complement proteins, oxidized LDL, endogenous mRNA, and extracellular ATP [7]. Both TLRs (via MyD88) and NLRs can trigger nuclear factor (NF)-κB nuclear translocation and subsequent transcription of IL-1β and IL-18 pro-proteins. Furthermore, activation of the NLRP3 inflammasome, composed of an NLR with pyrin domain, an adaptor protein, and Caspase-1, leads to proteolytic cleavage of these precursors and the release of inflammatory cytokines, particularly IL-1β and IL-18 [8]. 

In addition to their role in innate immunity and as responders to damage in the host, macrophages are integral to many homeostatic processes, particularly the phagocytosis of cellular debris and apoptotic cells, as well as the clearance of erythrocytes. This function is mediated by a host of cell-surface receptors and is immunologically inert—that is, it results in little or no production of inflammatory mediators by the macrophage [9]. Owing to their role in not only host defense, but also tissue repair, wound healing, and maintenance of homeostasis, macrophages have garnered much attention for their potential involvement in immune disorders, and AMD is no exception.

The presence of macrophages in or near AMD lesions, particularly in the drusen, Bruch’s membrane, choroid, and retina, is well-documented [10,11,12,13,14]. Immunostaining of CNV donor eyes showed the presence of CD68^+^ macrophages in Bruch’s membrane and the choroid; it has also been shown that the RPE expresses monocyte chemotactic protein (CCL2/MCP-1), which draws monocytes into the area [14]. Macrophages release tissue factor (TF), and both macrophages and the RPE produce vascular endothelial growth factor (VEGF) in CNV eyes [14]. VEGF is a potent mitogen, triggering the expansion of new blood vessels in patients showing CNV. Another study has shown that the RPE heavily secretes CCL2/MCP-1 in response to stimulation with IL-1β [15].

Macrophages have been thought of as classically or alternatively activated, with the designation M1 and M2, respectively. These phenotypes are extreme ends of a spectrum, with markedly different expression of receptors, chemokines, and cytokines [16]. M1 macrophages produce inflammatory cytokines and highly toxic reactive oxygen and nitrogen species in order to escalate the immune response and create a hostile environment for invading pathogens, compromised host cells, and tumor cells. The M1 phenotype can be triggered by diverse factors including IFN-γ, LPS, and TNF. They are CD86^+^ and are identified by flow cytometry as IL-12^hi^, IL-23^hi^, IL-10^lo^ [17]. 

M2 macrophages are CD163^+^ and are IL-12^lo^, IL-23^lo^, IL-10^hi^ [17]. They are anti-inflammatory and pro-angiogenic, and are activated by IL-4 and IL-10. The term “alternatively activated” covers a wide range of macrophage phenotypes that are involved in immunoregulation as well as tissue repair and remodeling. 

Macrophage polarization has a significant effect on the inflammatory environment and success of tissue remodeling following implantation with biologic scaffolds [18]. For example, implantation with one type of scaffold led to a relatively high proportion of M2 macrophages for 4 weeks following surgery, as measured by CD163 immunohistochemical staining, and resulted in constructive remodeling. Implantation with a second type led to M1 macrophage recruitment, measured by CD80 and CCR7 surface markers, and displayed chronic inflammation [18].

Preliminary evidence indicates that the macrophage population in the aging eye is dynamic, and differences are observed between healthy individuals in comparison to those with AMD [19]. Cao *et al.* (2011) extracted RNA from non-AMD and AMD tissue samples and compared *CXCL11* (secreted from M1 macrophages) and *CCL22* (from M2 macrophages) expression levels to determine the presence and ratio of M1 and M2 macrophages in the macular choroid. As compared to young (age <70 years) non-AMD samples, old non-AMD samples displayed elevated levels of M2 macrophages, suggesting a reparative environment established by the body in response to the normal oxidative stress of aging. However, the eyes of patients with AMD, and particularly geographic atrophy, showed significantly elevated levels of M1 macrophages, and a much higher M1:M2 ratio in the macula. Infiltration of M1 macrophages is found prior to the development of the AMD-like lesions in the outer retina of mice immunized with carboxyethylpyrrole (CEP) [20], a protein adduct generated from the oxidation of docosahexaenoic acid in the retina that has been shown to model CNV and GA [21,22,23]. This strongly suggests a link between classical M1 macrophage activation in the eye and the development of AMD. 

In August 2013, a small group of macrophage biologists met informally at the International Congress of Immunology and discussed the issue surrounding macrophage terminology, publishing an article regarding macrophage nomenclature in July 2014 [24]. Their stated goal is to remove ambiguity and provide a new naming system, based on a reproducible experimental standard, by using the culture conditions used to generate different macrophage phenotypes as the basis for the new nomenclature. Based on their proposed common framework for macrophage-activation nomenclature, M1 macrophages should be referred to as M(IFNγ) and/or M(LPS + IFNγ), and M2 macrophages should be labelled as M(IL-4) and/or M(GC) in AMD. 

### 2.2. Microglia

Microglia are resident tissue macrophages that accumulate subretinally with age in murine models [25]. In a healthy tissue environment, microglia resident to the inner retina are neuroprotective and secrete anti-inflammatory factors [26]. In the event of damage, they are the first-line phagocytic sentinels in the retina for neuronal homeostasis and innate immune defense [27]. When allostatic overload occurs and homeostasis is lost, microglia can act as potent mediators of runaway inflammation and persistent activation [28].

Microglia are normally present in the inner nuclear layer (INL) and inner plexiform layer (IPL), as confirmed by fast red labeling of CX3CR1 [29]. CX3CR1 is a receptor for CX3CL1, known as fractalkine, and variations in the gene leading to decreased function have been shown to correlate with AMD in human samples [30]. CX3CR1-positive cells coincided perfectly with CD18 expression, a microglial marker, which makes microglia the only cells in the retina that express CX3CR1 [29]. Individuals with the CX3CR1-M280 polymorphism displayed impaired microglial migration in response to CCL2, and *Cx3cr1*^−/−^ mice showed subretinal microglial accumulation and retinal degeneration associated with aging, laser injury, and albinism; additionally, microglial cells adjacent to the site of laser-induced CNV expressed VEGF, indicating that they may contribute to the initiation, maintenance, and progression of CNV [29]. Interestingly, subretinal microglial activation always preceded significant retinal degeneration, and this degeneration was ameliorated in albino mice raised in complete darkness, which resulted in limited microglial accumulation; furthermore, drusen-like bodies found in the subretinal space were composed of foamy microglial deposits following accumulation of intracellular lipids [29].

Time lapse confocal microscopy has shown that microglia are normally found in the inner retina in a quiescent state, with processes that extend in a randomized manner such that overall movement is limited. Upon damage to retinal tissue, however, microglia become activated and extend processes in the direction of insult, exhibiting a great degree of motility [31]. Microglia have been found in the subretinal space and outer nuclear layer (ONL) in AMD tissue samples containing rhodopsin-positive cytoplasmic inclusions, suggesting that rod photoreceptor debris had been phagocytosed [32]. A model of AMD pathogenesis has been proposed with microglia as one potential contributor to inflammation and immunity, with activated microglia generating some of the material composing drusen [33].

### 2.3. Complement Involvement

The complement system is another key component of innate immunity that comprises many small proteins primarily synthesized in the liver and found circulating in the blood. Macrophages are an important source of complement synthesis, second only to hepatocytes [34]. Components of this system can opsonize and lyse pathogenic organisms, as well as recruit and activate inflammatory cells (via the anaphylatoxins C3a and C5a) and modulate the inflammatory response [35]. The classical, alternative, and lectin pathways all converge on the formation of the membrane attack complex (MAC) which creates a pore in the cell membrane and results in cell lysis and death. 

Polymorphisms in the genes coding for complement regulatory proteins as well as complement effectors have shown a strong association with the development of AMD [36]. These polymorphisms reside within genes including *CFH*, *CFB/C2*, *C3* and *C5*, with *CFH* having shown the strongest association [37,38,39,40,41]. Notably, homozygosity for a sequence variant causing Y402H and I62V substitutions in *CFH* is strongly associated with risk of AMD. TLR3 and TLR4 have also shown associations [42,43], which may not be replicable [44,45].

Many complement components and regulatory proteins have been identified in drusen including all the components (C5–C9) that assemble to form the MAC [46]. In a recent *in vitro* study, cultured human RPE cells with high-risk CFH haplotypes (Y420H and 162V substitutions) were more susceptible than were low-risk haplotypes to complement-mediated attack in the face of bisretinoid accumulation secondary to photoreceptor phagocytosis [47].

Complement proteins and the MAC can trigger activation of the NLRP3 inflammasome, pointing to a link between complement activity and the activation of inflammatory cytokines including IL-1β and IL-18 [48]. The first detectable event upon sublytic MAC formation on the cell surface is Ca^2+^ influx and increased cytosolic Ca^2+^ concentration [49,50]. One study generated sublytic MAC formation in lung epithelial cells by exposing them to CD59 anti-serum and normal human serum (NHS). Cells exposed to CD59 anti-serum exhibited increased levels of activated Caspase-1 and IL-1β following MAC formation. This effect was prevented by blocking translation of the NLRP3 inflammasome using short hairpin RNA (shRNA), but not by blocking other NLRPs [51].

### 2.4. Pattern Recognition Receptors (PRRs)

PRRs are part of innate immunity; they are expressed by immune cells and host tissue. The PRRs include Toll-like receptors (TLRs), RIG-I-like receptors (RLRs), NOD-like receptors (NLRs), and C-type lectin receptors (CLRs) [6], which are encoded in the germ-line and hence are not a part of adaptive immunity. PRRs sense PAMPs, antigens which typically are essential to pathogen function and thus are highly conserved. The 10 TLRs identified in humans, for example, sense everything from lipopolysaccharide (LPS) to DNA, RNA, lipids, and viral envelope proteins. PRRs are essential to host immune defense in that they are very good at rapidly identifying and responding to infectious agents while sparing host cells [52].

TLRs are primarily expressed on the surface of innate immune cells including macrophages and dendritic cells; NLRs, on the other hand, are found within the cellular cytoplasm. Both TLRs and NLRs function in a similar fashion: leucine-rich repeat (LRR) motifs sense PAMPs and DAMPs, and upon interaction with downstream adaptor molecules, NF-κB is freed to enter the nucleus and up-regulate transcription of inflammatory cytokines including TNF-α, IL-1β, IL-6, and IL-18 [8]. NLRs can be found in the cell cytoplasm of epithelial cells, macrophages, and dendritic cells. NLRs have a caspase recruitment domain (CARD) at the amino terminus. Both NOD1 and NOD2 recruit the serine-threonine kinase RIPK2, which goes on to recruit and activate TAK1, followed by release of NF-κB into the nucleus and transcription of inflammatory cytokines [8].

### 2.5. NLRs: The NLRP3 Inflammasome

A subset of the NLRs contains a pyrin domain (PYD) at the amino terminus, in addition to the nucleotide-binding and oligomerization (NACHT) domain and carboxy-terminal LRR domain found in other NLRs [53]. These are known as NLRPs, or NACHT, LRR and PYD domains-containing proteins. PYD functions analogously to the TIR of TLRs, as well as the death domain of MyD88 and the CARD of other NLRs, in that it is responsible for homotypic protein-protein interaction. At present, 14 NLRPs are known to exist in humans (NLRP1-14). The NLRPs form complexes with other proteins to create a structure called the inflammasome, consisting of an NLRP, an adaptor protein called PYCARD/ASC, and caspase-1 [54]. Caspase-1 is known to be involved in activation of inflammation and apoptosis in many cells by cleaving the zymogens IL-1β and IL-18 and resulting in the release of active inflammatory cytokines.

Of particular interest to AMD researchers of late is NLRP3, also known as NALP3 or cryopyrin. In stressed cells, NLRP3 proteins dimerize and recruit PYCARD/ASC associated with caspase-1 pro-enzyme through their respective CARD domains, forming a structure called the NLRP3 inflammasome. Auto-catalytic cleavage results in active caspase-1, which then cleaves pro-IL-1β and pro-IL-18 into their mature forms [54].

**Figure 1:**
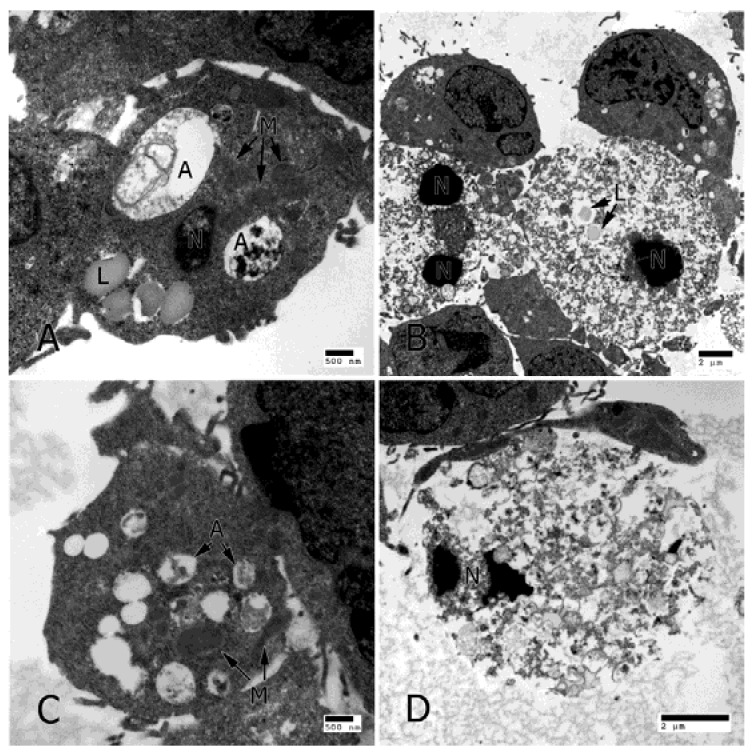
Electron micrographs showing pyroptosis in mouse retinal stem cells (RSC) in response to *in vitro* application of IL-1β and IL-18 (A). IL-1β induced mitochondrial (M) damage, autophagy (**A**), glycogen accumulation (not labeled), lipid droplets (L), and nuclear condensation (N); (**B**) IL-1β induced advanced disintegration of cytoplasm in RSCs; (**C**) IL-18 induced mitochondrial (M) damage and autophagy (A); (**D**) IL-18 induced advanced cytoplasmic disintegration and nuclear condensation (N).

The NLRP3 inflammasome promotes maturation of IL-1β and IL-18, and significantly elevated levels of NLRP3, IL-1β, and IL-18 expression have been found in AMD macular lesions [55,56,57]. Interestingly, *in vitro* chemical disruption of lysosomes can trigger caspase-1 activation, IL-1β production and RPE death; this lysosomal destabilization is capspase-1 dependent, characteristic of pyroptosis [53]. In fact, electron microscopy reveals that mouse retinal stem cells (RSC) exposed to IL-1β and IL-18 exhibit classic signs of ultrastructural cellular damage, including formation of autophagic vesicles, mitochondrial breakdown, accumulation of glycogen and lipid droplets, and in some cells pyroptosis can be observed (Figure 1). siRNA knockdown of NLRP3 inhibited IL-1β and IL-18 production in RPE cells in response to stimulation with TNF-α [58]. IL-1β and IL-18 are elevated in patients with dry AMD that are homozygous for the CFH Y402H risk variant; however, cytokine levels were not found to be related to drusen area or choroidal thickness [59]. In addition to CEP, an AMD biomarker found in drusen [60], complement component C1q has been shown to activate the inflammasome; drusen themselves cause NLRP3 activation and secretion of IL-1β and IL-18 in peripheral blood mononuclear cells (PMBCs) [61].

Recently, it was discovered that accumulation of *Alu* RNA encoded by small repetitive *Alu* elements in the genome is tied to degeneration of RPE cells [62]. Knockdown of DICER1, which, in addition to its other functions, is responsible for the degradation of toxic *Alu* transcripts, resulted in RPE degeneration which was blocked by antisense oligonucleotides that targeted *Alu* RNA sequences [62]. While it is not yet known how a DICER1 deficit occurs *in vivo*, Kaneko *et al.* (2011) have suggested oxidative stress as the culprit, showing that hydrogen peroxide downregulated DICER1 in human RPE cells. It is now known that *Alu* RNA exposure or DICER1 deficit can directly prime the NLRP3 inflammasome, and resulting RPE degeneration can be blocked by inhibition of inflammasome components or IL-18 [55]. Interestingly, *Alu* RNA-induced inflammasome priming and RPE degeneration depends on NF-κB and purinoreceptor P2X7, not TLR signaling [63].

NLRP3-processed cytokines play a role in the production of IL-17. IL-1β and IL-18 have both been shown to promote secretion of IL-17 by γδ and CD4^+^ Th17 cells [64]. Furthermore, IL-17 production by γδ T cells has been implicated in an activation loop whereby Th17 cells are stimulated to produce even more IL-17 [65]. A model has been proposed where inflammasome activation and subsequent IL-1β and IL-18 production result in elevated levels of IL-17 secretion and auto-immunity [66].

## 3. IL-17 in AMD

The first insight into the role of IL-17 in AMD came with the observation that increased serum levels of IL-17 are found in patients with wet AMD compared to a healthy cohort [67]. Another study has since replicated this finding and also identified increased circulating levels of IL-1β, IL-1α, IL-4, IL-5, IL-10, and IL-13 [68]. Liu *et al.* (2011) demonstrated that CD3^+^CD4^+^ T cells extracted from peripheral blood mononuclear cells (PBMCs) actively produced IL-17 when co-cultured with complement component C5a *in vitro*. Importantly, Liu *et al.* (2011) determined that C5a-mediated induction of IL-17 in T cells was dependent on both the presence of CD14^+^ monocytes in the culture system and these monocytes’ ability to both upregulate the co-stimulatory proteins B7.1/B7.2 and secrete IL-1β and IL-6. Antibodies that blocked either the B7-CD28 interaction or that neutralized IL-1β or IL-6 in the supernatant extinguished the production of IL-17.

**Figure 2:**
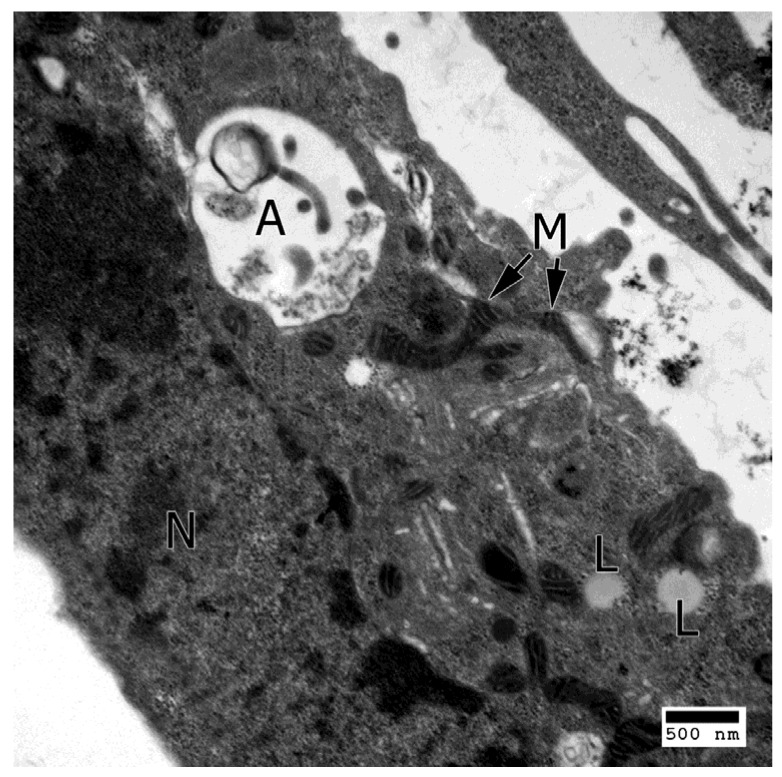
Electron micrograph showing the effects of *in vitro* application of IL-17 to ARPE-19 cells. IL-17 induced mitochondrial (M) damage as well as autophagosome (A) formation. Nuclear membrane appears compromised (N), chromatin is diffused and heterochromatin distributed along the nuclear membrane. Lipid droplets (L) are observed in the cytoplasm.

Recently published data have further delineated a retinotoxic effect of IL-17 in the pathogenesis of AMD [69]. PBMCs extracted from AMD patients exhibit hypomethylation of the IL-17RC promoter has been reported in one study [70], which coincides with enhanced expression levels of both IL-17A and IL-17RC in AMD maculae when compared with both healthy maculae and the peripheral retinas of AMD-afflicted eyes [69]. IL-17A is damaging to ARPE-19 cells *in vitro*—causing activation of caspases and resulting in mitochondrial dysfunction and cell death (Figure 2)—and blockade of IL-17 signaling in an animal model significantly improves and reverses development of AMD-like lesions [69]. 

Two IL-17 polymorphisms, rs2275913 and rs3748067, have been associated with AMD [71]. Both SNPs were shown to influence production of IL-17 from PBMCs upon stimulation with phytohemagglutinin (PHA) in a genotype-dependent manner, with cell populations that were homozygous for higher-risk alleles producing greater amounts of IL-17 compared to either heterozygotes or those homozygous for wildtype. Importantly, it is not clear whether patients with systemic autoimmune diseases were excluded from this study, or if SNPs would have significance in non-Chinese populations. 

## 4. Apoptosis, Pyroptosis, Necroptosis and Autophagy in AMD

**Figure 3:**
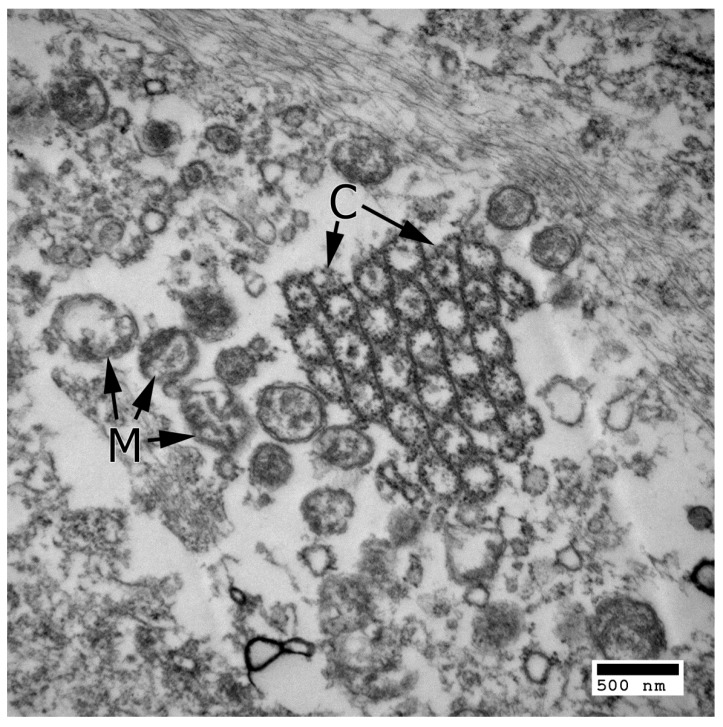
Electron micrograph from a patient with advanced AMD showing extranuclear DNA. Extranuclear chromatin (C) and mitochondrial disintegration (M) is observed. Chromatin strands form vesicles that clump together to form a lattice.

In degenerative diseases such as AMD, the disintegration of cells and their organelles results in a loss of function and the accumulation of cellular byproducts of intermediate substances and compounds in the cytoplasm and extracellular space. As has been seen in clinical cases, the degenerative process is often associated with mitochondrial dysfunction (Figure 1, Figure 2 and Figure 3), and thus low availability of energy [72]. Mitochondria are known to play a critical role in cellular death, particularly in the intrinsic pathway of apoptosis [73], and have been linked to activation of the inflammasome and presumably pyroptosis [74]. Abnormal ultrastructural changes may expand to nuclei, such as DNA leakage (Figure 3) and fragmentation [69]. Given the previously discussed literature implicating inflammasome activation in AMD, the issue that needs to be addressed is what types of cell death are induced by inflammation and taking place in AMD.

### 4.1. Apoptosis

Apoptosis is the best characterized form of programmed cell death; it was first coined in 1972 by Kerr *et al.* [75]. The biochemical pathways involved in apoptosis typically involve a set of caspases, or cysteine-aspartic proteases [76]. The apoptotic process has distinctive morphological features that distinguish it from other types of eukaryotic cell death; it includes rounding of cells and retraction of pseudopods and irreversible condensation of chromatin (pyknosis). The nucleus itself fragments (karyorrhexis) and blebbing is observed in the plasma membrane. What is left of the cell is then engulfed by resident phagocytes [77]. One way in which phagocytosis is mediated is through the expression of phosphatidyl serine on the cell surface [78,79]. Apoptosis is inherently anti-inflammatory in nature; upon phagocytosis of apoptotic cells by macrophages, production of TGF-β and prostaglandin E2 is induced and inflammation is resolved [80]. Apoptosis is the type of cell death most invoked in the AMD literature; however, our ultrastructural observations support the involvement of other types such as pyroptosis where the pathways involved are initiated by inflammation and require much less cellular energy to proceed than apoptosis [81]. The cell death observed is marked by the presence of many autophagosomes. There is also the notion that an apoptotic process may switch to other death types, thus these death types may appear to share initial events in their pathways [81].

### 4.2. Pyroptosis

The term pyroptosis was first proposed to describe an alternative cell death pathway linked to inflammation [82]. It is entirely dependent on activation of caspase-1 [83], which as discussed previously is one of the key components of the NLRP3 inflammasome [54]. Pyroptosis involves the secretion of inflammatory cytokines including IL-1β and IL-18. A cell undergoing this form of cell death forms pores in the cell membrane and undergoes rapid lysis, releasing cytosolic contents including inflammatory cytokines into the extracellular space [84]. Thus, pyroptosis is not a neat, orderly shrinking and phagocytosis of cells in the way that apoptosis is, but occurs quickly and leads to inflammation (Figure 1B,D). Our knowledge now of the amount of inflammation that occurs within AMD’s phenotypes, as well as the involvement of the NLRP3 inflammasome, suggests possible pyroptosis rather than apoptosis as a death pathway.

### 4.3. Necroptosis

Necroptosis is a term for regulated cell death, which resembles passive necrosis but is actually mediated by programmed biochemical pathways within the cell. It is a form of non-apoptotic cell death that is mediated by the kinase activity of RIP1 and RIP3 [85,86], and is caspase-independent—in fact, caspase inhibitors cause cells exposed to TNF-α to undergo necroptosis as opposed to apoptosis [87]. Necroptosis induced in cells by TNF-α and caspase inhibition was blocked by a molecule called necrostatin-1, first suggesting that this type of cell death is actually a regulated program executed by cells, and not the type of passive necrosis seen in cells under extreme physiological stress [88]. During necroptosis, the cell and its organelles swell, and the plasma membrane ruptures and releases its contents [77]. This rupturing is presumed to release DAMPs into the surrounding tissue, though the physiological importance of necroptosis has not been fully elucidated [89].

### 4.4. Autophagy

Autophagy is an evolutionary conserved mechanism by which the mitochondria sequesters and expels xenobiotic material into the cytoplasm. The mitochondria directly donates its membrane during the formation of autophagosomic vesicles, which then turn into lysosomes [90]. Autophagosomes possess a variable number of enclosed membranes; because the mitochondrial population within a cell is heterogeneous, each mitochondrion varies in its rate of autophagosome formation. Autophagy can be thought of as a defense mechanism by which the mitochondrion gives up a part of itself in the form of a vesicle to survive an influx of xenobiotics. However, the continued exposure causes mitochondrial depletion, cellular dedifferentiation, and the eventual death of the cell. Autophagy as described here is a mitochondrial phenomenon that may save the cell or destroy it depending on the duration and the intensity of the insult. Autophagic cell death is easily distinguishable by transmission electron microscopy through massive autophagic vacuolization of the cytoplasm and the absence of chromatin condensation [77]. Some cytokines are inducers of autophagy. According to our experimental work, cytokines such as IL-1β and IL-17 can trigger autophagy and cell death [69,91]. Thus, chronic inflammation in the retina may induce AMD through autophagy (Figure 1 and Figure 2). ARPE-19 cells exposed to IL-17 showed nuclear disintegration in addition to autophagy (Figure 2). Cytosolic DNA from the damaged cellular nuclei is a potent DAMP that has been shown to upregulate expression of several inflammatory cytokines [92], which may contribute to the inflammatory environment observed in AMD.

## 5. Conclusions

The goals of research on AMD are to establish effective prevention practices for healthy individuals and efficient clinical management of diseased individuals. However, fulfilling these two goals requires a new synthesis of the available AMD data into a disease model that will serve as the road map to actualizing these goals. In this review, we evaluated the literature in relation to inflammation and cell death, and embarked on constructing an AMD etiological model.

When considering the classification of AMD pathogenesis, we are hesitant to label this disease an immune disorder. Indeed, the data all point to up-regulation, overexpression, and ectopic expression of inflammatory cytokines—IL-1β, IL-17, IL-18—inflammasome, complement, macrophages and microglia that are closely related to the innate immune system. However, at its heart, AMD is a disease associated with aging and it is in those ways that AMD differs from aging that we may uncover the etiology [93]. Aging is an incompletely understood process and offers many confounders into the analysis. One must consider simultaneously the interplay of genetic predisposition with environmental exposure amidst a backdrop comprised of the immune response to these factors. At the same time, one must consider that not all individuals experience aging in the same way and to complicate matters, one must also consider the aging of the immune system itself. 

Where AMD differs from most immune disorders is in the factors involved. Typical complement disorders include C3 deficiency, in which individuals are susceptible to infections such as *N. meningiditis*. Adaptive immune disease may be thought of as underactive, such as in HIV infection or more rare genetic causes, or overactive, such as in autoimmune diseases like psoriasis, multiple sclerosis, and autoimmune uveitis. AMD, on the contrary, has a peculiar association with inflammatory cytokines that is usually much milder compared to uveitis. We point specifically to our recent finding that there are increased levels of IL-17—both protein and mRNA—within AMD maculae [69] and that the ultrastructure of some IL-17 stimulated ARPE-19 cells clearly displays autophagy and mitochondrial damage (Figure 2). IL-17 is a potent cytokine produced by Th17 cells which up-regulates pro-inflammatory cytokines such as IL-6 and IL-8, a chemotactic factor for neutrophil recruitment. Given the paucity or lack of Th17 cells and neutrophils within AMD lesions, this begs the question as to what these pro-inflammatory factors do in the absence of inflammation. 

We posit, particularly in light of our findings and those of our peers who identified a role for the inflammasome system [55,58,61,94] in AMD, that AMD may indeed represent an age-related susceptibility to aberrant innate immune activation based on acquisition of foreign-like structural motifs. The innate immune system is able to facilitate autosensitization [95,96,97]. That is, as a result of age-related insults—be they metabolic, biochemical, *etc.*—endogenous proteins acquire patterns recognized by PRRs as foreign despite being nothing of the sort, initiating a cycle where the immune system becomes chronically activated, leading to further release of DAMPs and ultimately cell death. In this way, AMD may be viewed as a disease of the innate immune system. Thus, AMD, in contrast to diseases of adaptive immunity, would require acquired changes to substrates over time due to the immutable, germ-line encoded nature of the PRRs. This stands in sharp contrast to errors in the adaptive immune system, which are governed by genetic machinery designed by evolution to encourage active identification of new non-self motifs. The activation of innate immunity results in damage to retinal tissue and cell death mediated by inflammatory cytokines and potentially Caspase-1 mediated pyroptosis, with excessive autophagy (Figure 1 and Figure 2), extranuclear DNA (Figure 3), and mitochondrial dysfunction (Figure 1, Figure 2 and Figure 3) present and observed by EM. Because the study of different means of cell death and their physiological importance is an emerging field, future research in AMD can use these concepts to elucidate how photoreceptor and RPE death occurs and develop novel targets for treatment going forward. 
